# Supplementary material for: A toolkit for converting Gal4 into LexA and Flippase transgenes in Drosophila
Source: G3 (Bethesda). 2023 Jan 6;13(3):jkad003. doi: 10.1093/g3journal/jkad003 (PMC9997562; doi:10.1093/g3journal/jkad003)
Supplement: jkad003_Supplementary_Data [file jkad003_supplementary_data.zip › Figure_S1_G3-2022-404030.pdf]

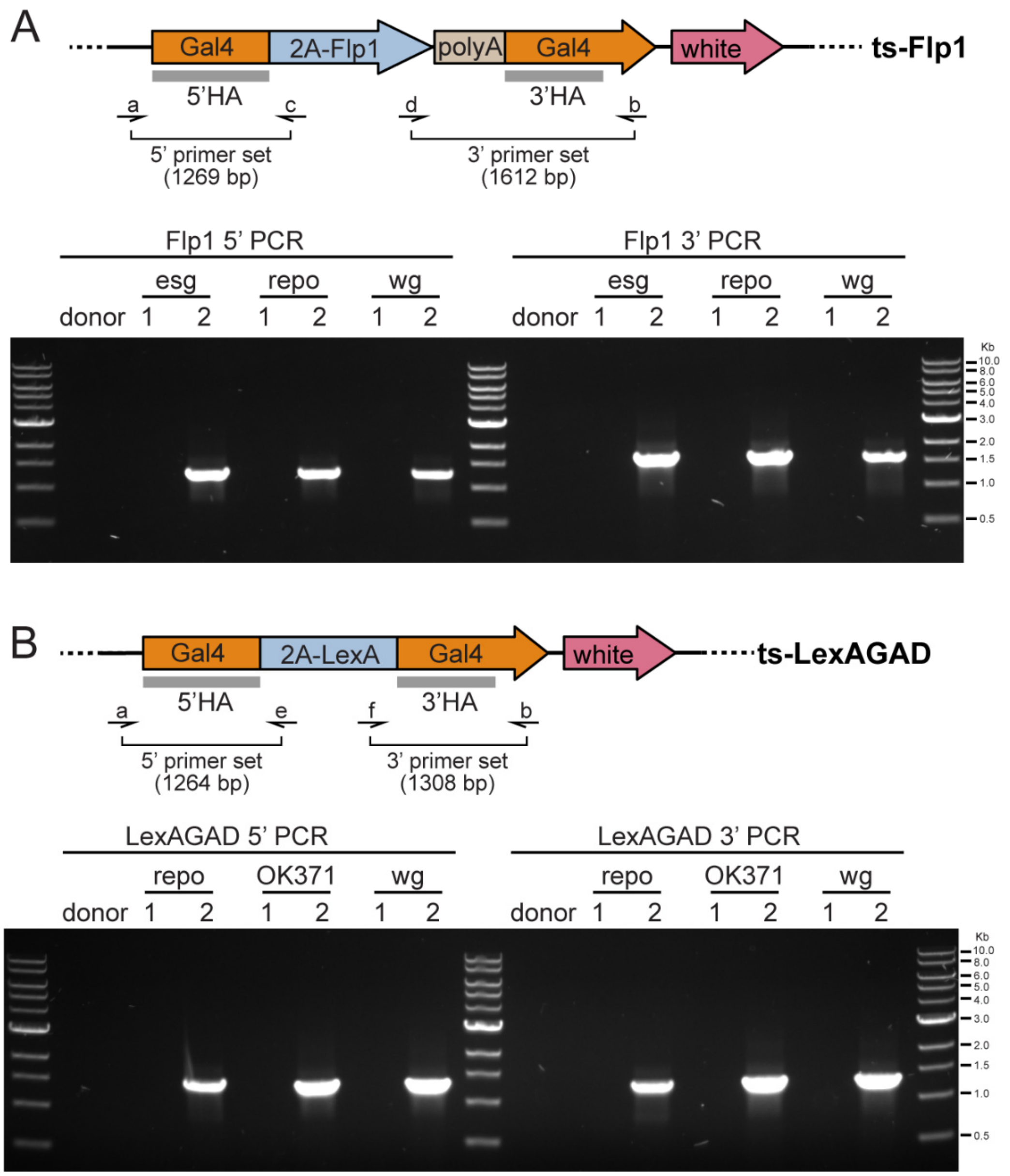

Figure S1. Validation of converted LexAGAD and Flp lines by genomic PCR

(A) Diagram of converted Flp transgene and genomic PCR results for *esg*, *repo*, and *wg* lines. The positions of 5' and 3' homology arms (HAs), binding locations of PCR primers, and expected sizes of PCR products are indicated in the diagram. The DNA gel shows PCR results of the Flp donor line, the original Gal4 (1), and the converted Flp (2).

(B) Diagram of converted LexAGAD transgene and genomic PCR results for *repo*, *OK371*, and *wg* lines. The diagram and PCR results are labeled similarly to (A).

The primers used for PCR amplifications are (a) CTTGAAGCAAGCCTCCTGAAAG; (b) AGTGGTATTAAACATCCCTGTAGTG; (c) TGACGCACCAACACCTTTG; (d) CAGGAGGTTCTGGATTACCTGAG; (e) GAGAGCCTTCATTGGATCTTCTAC; (f) ACCATCTACCACGGTATCATTGAG.
