# Supplementary material for: A toolkit for converting Gal4 into LexA and Flippase transgenes in Drosophila
Source: G3 (Bethesda). 2023 Jan 6;13(3):jkad003. doi: 10.1093/g3journal/jkad003 (PMC9997562; doi:10.1093/g3journal/jkad003)
Supplement: jkad003_Supplementary_Data [file jkad003_supplementary_data.zip › Figure_S2_G3-2022-404030.pdf]

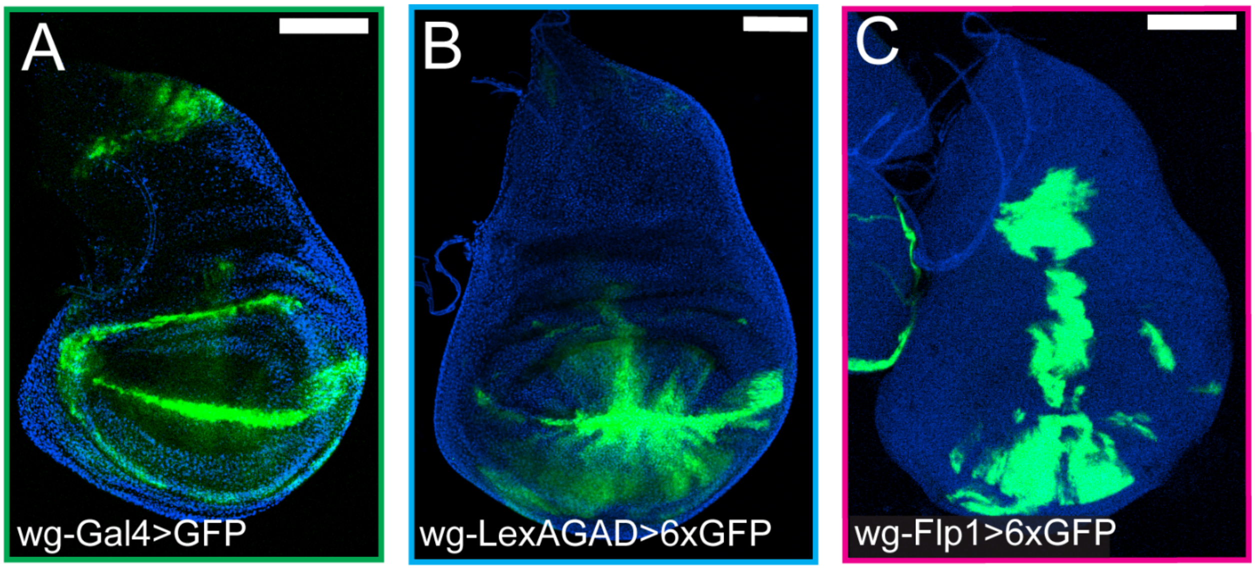

Figure S2. Comparison of *wg-Gal4*, *wg-LexAGAD*, and *wg-Flp* lines in the wing disc

(A-C) Activity patterns of *wg-Gal4* (A), *wg-LexAGAD* (B), and *wg-Flp1* (C) in a 3rd instar wing imaginal disc. GFP is in green; DAPI staining is in blue.

Refer to Table 1 for reporter lines used. Scale bar: 100 μm in all panels.
